# Assessment of quality of antenatal care services and associated factors in Malawi: Insights from a nationwide household survey

**DOI:** 10.1371/journal.pone.0305294

**Published:** 2024-06-12

**Authors:** Chrispin Mandiwa, Bernadetta Namondwe

**Affiliations:** 1 PhD Program in Global Health and Health Security, College of Public Health, Taipei Medical University, Taipei, Taiwan; 2 Elizabeth Glaser Pediatric AIDS Foundation (EGPAF), Lilongwe, Malawi; Kwame Nkrumah University of Science and Technology College of Health Sciences, GHANA

## Abstract

**Introduction:**

Provision of quality antenatal care (ANC) to pregnant women is essential for reducing maternal and newborn mortality. ANC provides an opportunity for early identification of conditions that increase the risk of adverse pregnancy outcomes. However, there is limited evidence regarding the quality of ANC received by women in Malawi. This study aimed to assess the quality of ANC and associated factors in Malawi.

**Materials and methods:**

National representative data from the 2019–2020 Malawi Multiple Indicator Cluster Survey was used for this cross–sectional study. A total of 6,287 weighted sample of women aged 15 to 49 years who had a live birth and received ANC at least once within two years preceding the survey were included in the analysis. Descriptive statistics were used to estimate the magnitude of quality ANC and multivariable logistic regression was computed to identify associated factors.

**Results:**

Of the 6,287 women, only 12.6% (95% CI: 11.4–13.9) received quality ANC. The likelihood of receiving quality ANC was significantly higher among women who had four to seven ANC contacts (AOR = 2.10; 95% CI: 1.79–2.49), made at least eight ANC contacts (AOR  =  3.40; 95% CI: 1.90–6.09) and started ANC within the first trimester (AOR  =  1.30; 95% CI: 1.10–1.53). On the other hand, women who had only primary education (AOR = 0.62; 95% CI:0.48–0.82) and had five or more births (AOR  =  0.56; 95% CI: 0.40–0.78) were less likely to receive quality ANC.

**Conclusion:**

The findings reveal that quality of ANC in Malawi is low. These findings suggest the need for targeted interventions aimed at improving access to and utilization of ANC services among women with lower education and higher parity. Strengthening efforts to promote early ANC initiation and increasing the number of ANC contacts could significantly enhance the quality of ANC received by women in Malawi.

## Introduction

Maternal mortality remains a major problem in many parts of the world. Estimates from 2020 show that 287,000 or more women across the world die each year from pregnancy and childbirth related complications [1]. Similarly, in 2021, approximately 1.9 million stillbirths were reported globally [2]. Most of these deaths occurred in sub-Saharan Africa [1,2]. Existing evidence suggests that providing quality antenatal care (ANC) services to pregnant women can help to avert many of the life-threatening pregnancy complications and reduce maternal and neonatal deaths in low- and middle-income countries (LMICs) [3–5]. In recognition of the importance of ANC in improving maternal and child health, many global initiatives have focused on promoting ANC services. For instance, the Safe Motherhood Initiative (a universal campaign, which wanted to reduce maternal mortality by half between 1987 and 2000) identified ANC as one of the four main pillars (alongside family planning, safe delivery and postnatal care) to reducing maternal mortality in developing countries [6]. The Millennium Development Goals (MDG) included ANC as one of the critical components for accelerating progress towards improving maternal health [7]. In 2015, the Sustainable Development Goals (SDG) added ANC as a tracer indicator for global monitoring of maternal health services for the universal health coverage [8]. The inclusion of ANC in these three major global campaigns underscores its importance in improving maternal and child health.

The link between ANC and maternal health has been widely discussed in literature [9–11]. ANC enhances maternal and child health by providing important opportunities for early detection and management of conditions such as maternal anaemia, preeclampsia, gestational diabetes mellitus and sexually transmitted infections that increase risk of adverse pregnancy outcomes [12]. If these conditions are not detected and managed properly during prenatal period, they may result into unfavourable pregnancy outcomes including preterm delivery, low birth weight, stillbirth, macrosomia, birth defects, abortion and maternal complications [13,14]. ANC also provides a chance for reaching pregnant women with a number of preventive interventions that may be crucial to their health and that of their babies [4]. For example, ANC provides an important entry point for offering HIV testing and medications to prevent mother to child transmission (PMTCT) of HIV. During ANC, pregnant women are also provided with important information on healthy behaviours during pregnancy, birth preparedness, institutional delivery and other information essential for improving perinatal outcomes and child survival [15]. This suggests that the role of ANC in maternal health is to ensure a healthy mother and baby at the end of pregnancy.

In 2016, the World Health Organization (WHO) modified its recommended minimum number of ANC contacts from four to eight contacts at 12, 20, 26, 30, 34, 36, 38 and 40 weeks of gestation following recent evidence that increased number of contacts between an expectant woman and a skilled health provider reduced perinatal mortality and improved women’s experience of care [4].The 2016 WHO ANC model has also provided universal and context-specific recommendations on contents of ANC that must be provided to pregnant women during ANC visit. The 2016 WHO ANC model emphasises the importance of quality of care and recommend that during each ANC contact, a pregnant woman should have blood pressure measured, urine sample collected for analysis, blood sample collected for examination, given doses of tetanus vaccine and prophylaxis antimalarial drugs according to schedule among others [4].

Malawi has made significant improvements in maternal health indicators over the last three decades. The proportion of pregnant women who received ANC from a skilled provider has increased from 90% in 1992 to 97% in 2019–2020 [16,17]. That same period, health facility deliveries have increased from 55% in 1992 to 97% in 2019–2020 [16,17]. Despite this remarkable progress in ANC coverage, maternal mortality ratio (MMR) still remains unacceptability high. As of 2020, the MMR was estimated at 381 per 100,000 live births [1]. This puts into question the quality of ANC offered to pregnant women in health facilities.

Poor-quality ANC services can have devastating consequences for maternal and infant health outcomes. It can lead to increased maternal and neonatal mortality rates, as well as complications during childbirth such as preeclampsia [18]. Additionally, poor-quality ANC may result in adverse pregnancy outcomes including preterm birth [19], low birth weight [20], stillbirth [2], and neonatal death [21]. Thus, poor-quality ANC can have long-term health consequences for both mothers and infants, contributing significantly to the burden of disease in communities.

Most studies conducted in Malawi have mainly focused on timing and frequency of ANC contacts [22,23], little attention has been paid to the quality and content of ANC pregnant women receive in health facilities. A previous study conducted in Malawi found that only 11% of pregnant women received quality ANC [24]. However, the study was based upon data collected over a decade ago and the results cannot be used to understand the current situation of ANC services provision in Malawi because the contents of ANC might have changed over time. In light of the aforementioned gap and the need for updated data, this study aimed to assess the quality of ANC services that pregnant women receive in health facilities and examine factors associated with receiving quality ANC in Malawi using data from the most recent nationally representative survey. We sought to identify the extent to which pregnant women receive quality ANC in health facilities and to provide evidence-based recommendations to enhance provision of quality ANC to pregnant women in Malawi.

## Materials and methods

### Data source, study design and setting

Data for the present cross-sectional study were extracted from the Malawi Multiple Indicator Cluster Survey (MICS) 2019–2020, a nationally representative household survey. The MICS 2019–2020 was the sixth-round survey and the most recent in Malawi. The objective of the MICS was to provide information on indicators for monitoring the welfare of women and children at the national level for rural and urban areas across all the 28 districts in Malawi. The MICS also provide information on indicators for monitoring progress of attainment of the SDGs and Malawi Growth and Development Strategy (MGDS) and other development programmes. The 2019–2020 MICS was conducted between December 2019 and August 2020 by the National Statistical Office (NSO), Malawi. Technical and financial support was provided by the United Nations Children’s Fund (UNICEF) and other international development organisations [17].

### Sampling method and study participants

The MICS 2019–2020 followed a two-stage sample design for the selection of the survey sample. The first stage involved systematic selection of enumeration areas (EAs) according to probability proportional to size. The second stage involved the listing of households within each selected EA, followed by a systematic random selection of households. In total, 25,419 households were interviewed, giving a response rate of 99 percent [17]. In the households interviewed, 25,626 women aged 15–49 years were eligible for interviews and of these 24,543 were interviewed using the individual woman’s questionnaire which captured data on a wide range of health indicators, including information about the elements of care provided during pregnancy, the timing of the first ANC contact, the number of ANC contacts made during the last pregnancy and women’s socio-economic and demographic characteristics [17]. Of the 24,543 interviewed women, we excluded 18,120 women who had no live birth in the last two years preceding the survey. We also excluded 136 women who did not attend ANC. Thus, our analysis was conducted on a weighted sample of 6,287 women who had a live birth and received ANC at least once within the last two years preceding the survey. The two-year timeframe was chosen to provide a comprehensive understanding of recent ANC service provision. By focusing on this timeframe, we aimed to reduce recall bias and ensure a more accurate assessment of the factors associated with the quality of ANC in Malawi.

### Measurement of variables

#### Outcome variable

The dependent variable for this study was quality of ANC. During the interviews, women were asked if they had received the following components of ANC services during pregnancy: (1) blood pressure measurement, (2) urine sample taken, (3) blood sample taken, (4) given three or more doses of sulfadoxine and pyrimethamine (SP), and (5) received at least two doses of tetanus vaccine. Responses were recorded as ’yes’ or ’no’ for each component. Women who reported receiving all five ANC services were categorized as having received quality ANC, while those who did not receive all components were classified as having received substandard ANC.

#### Independent variables

The selection of potential independent variables for this study was based on previous studies that examined quality of ANC and associated factors in LMICs [25–29]. The independent variables included were: age of the woman which was collected as an integer during the survey and was recoded into three categories for this study: 15–19, 20–34 and 35–49 years, area of residence (rural, urban), region of residence (northern, central, southern), marital status (never, currently and formerly married), exposure to radio (not at all, less than once a week, at least once a week), education level (no formal education, primary and secondary school or above), covered by health insurance (yes, no), number of ANC contacts categorized into inadequate (1–3 ANC contacts), moderate (4–7 contacts) and adequate (≥8 ANC contacts), parity (one, two to four, five or more), timing of first ANC initiation in months (categorized as within the first three months and after three months) and wealth index (categorized into three groups: poor, medium and rich). The wealth index was computed using the principal component analysis (PCA) method which has been described in detail elsewhere [30,31]. In brief, the wealth index was constructed through weighting each asset or dwelling characteristic and standardizing the resulting wealth score to a standard normal distribution. The index was provided as a wealth score separated into quintiles (poorest, second, middle, fourth, richest) in the MICS dataset. This study further regrouped this variable into poor (by combining poorest and second into poor), medium and rich (by combining richer and richest into rich) for ease of ordinal interpretation.

### Data analysis

Data were extracted, cleaned, and analysed using complex samples analysis of the Statistical Package for Social Sciences (SPSS version 23, IBM Corp., Armonk, NY, USA). To account for the multistage design of the survey, as well as to address issues such as non-responses and geographical strata selection variability, the woman’s sample weighting, primary sampling unit (PSU), and strata variables were used to create a complex samples analysis plan which was used throughout all the analyses. Descriptive analysis was used to describe the characteristics of the study participants and the results were presented as frequencies and percentages. Cross-tabulation and χ^2^ tests were used to examine the distribution of the outcome variable across the independent variables. Corresponding *p*-values from the χ^2^ test were used to determine the statistically significant relationship between the dependent variable and the independent variables. All variables with a *p*-value of ≤0.25 from the χ^2^ test were included in the final multivariable logistic regression model to determine and quantify the factors associated with quality of ANC [32]. Adjusted odds ratios with their respective 95% confidence intervals were calculated. A *p*-value of < 0.05 was considered significant. All tests were two-sided. Variables included in the final multivariable logistic regression model were assessed for multicollinearity using the variance inflation factor (VIF). All observed VIF values were found to be less than 10, indicating the absence of multicollinearity.

### Ethics statement

The present study was based on secondary analysis of publicly available dataset from the MICS 2019–2020 and therefore no further ethical approval was necessary. However, the authors were granted permission by the UNICEF MICS Team to use the data. Moreover, the MICS 2019–2020 report indicated that verbal consent was obtained from all research participants before interviews were conducted. Informed consent was obtained from parents or legal guardians of participants under 18 years (minors). Furthermore, all respondents were assured of voluntary participation, confidentiality, anonymity of information, and free will to withdraw from the study at any point.

## Results

### Description of the study participants

Table 1 displays the characteristics of the participants who were included in the analysis. Almost all the participants (99.5%) were not covered by health insurance and majority of the participants were rural dwellers (86.5%), were currently married (79.4%), had only primary education (68.2%) and were aged between 20 years and 34 years (69.7%). More than half of the participants (54.1%) had no exposure to radio, (30.9%) had given birth to one child. Almost half of the participants (48.6%) were from poor households and 45.1% were from southern region of Malawi. Half of the participants (50.6%) had made four to seven ANC contacts. Regarding ANC timing, only 27.8% of women started ANC within the first trimester.

**Table 1:** Characteristics of women who had a live birth and received ANC at least once in the last two years preceding the survey in Malawi (n = 6,287).

Variables	Weighted frequency	%
**Age(years)**		
15–19	963	15.3
20–34	4384	69.7
35–49	940	15.0
**Area of residence**		
Rural	5441	86.5
Urban	846	13.5
**Region**		
Northern	692	11.0
Central	2760	43.9
Southern	2835	45.1
**Marital status**		
Never married	367	5.8
Currently married	4989	79.4
Formerly married	931	14.8
**Education**		
None	527	8.4
Primary	4285	68.2
Secondary and above	1475	23.5
**Exposure to radio**		
Not all	3399	54.1
At least once a week	780	12.4
Less than once a week	2108	33.5
**Parity**		
1	1942	30.9
2–4	3181	50.6
≥5	1164	18.5
**Household wealth index**		
Poor	3059	48.6
Middle	1106	17.6
Rich	2122	33.8
**Covered by health insurance**		
Yes	6257	99.5
No	30	0.5
**Timing of first ANC contact**		
≤3 months	1746	27.8
>3	4541	72.2
**Number of ANC contacts**		
1–3	3041	48.4
4–7	3181	50.6
≥8	65	1.0

Table 2 presents the results of χ^2^ test of independence. All variables including age, area of residence, marital status, education, exposure to radio, parity, wealth index, timing of ANC visits and number of ANC contacts were associated with quality of ANC (*p*<0.05), except health insurance (χ^2^ **=** 3.78, *p* = 0.052) and region of residence (χ^2^ **=** 2.70, *p* = 0.259).

**Table 2:** Relationship between quality of ANC and independent variables.

Variables	Received quality ANC					
	No		Yes			
	n	%	n	%	χ^2^	*P*-value
**Age(years)**					13.90	0.001*
15–19	823	85.5	140	14.5		
20–34	3817	87.1	567	12.9		
35–49	854	90.9	86	9.1		
**Area of residence**					15.00	<0.001*
Rural	4790	88.0	651	12.0		
Urban	704	83.2	142	16.8		
**Region**					2.70	0.259
Northern	596	86.1	96	13.9		
Central	2400	87.0	360	13.0		
Southern	2498	88.1	337	11.9		
**Marital status**					7.79	0.020*
Never married	313	85.3	54	14.7		
Currently married	4343	87.1	646	12.9		
Formerly married	838	90.0	93	10.0		
**Education**					40.93	<0.001*
None	450	85.4	77	14.6		
Primary	3821	89.2	464	10.8		
Secondary and above	1224	82.9	252	17.1		
**Exposure to radio**					8.72	0.013*
Not all	3006	88.4	393	11.6		
At least once a week	682	87.3	99	12.7		
Less than once a week	1806	85.7	301	14.3		
**Parity**					38.48	<0.001*
1	1635	84.2	307	15.8		
2–4	2791	87.7	390	12.3		
≥5	1068	91.8	96	8.2		
**Household wealth index**					10.97	0.004*
Poor	2702	88.4	356	11.6		
Middle	978	88.4	128	11.6		
Rich	1814	85.4	309	14.6		
**Covered by health insurance**					3.78	0.052
Yes	5471	87.4	786	12.6		
No	23	74.2	7	25.8		
**Timing of first ANC contact**					32.56	<0.001*
≤3 months	1458	83.5	288	16.5		
>3	4036	88.9	505	11.1		
**Number of ANC contacts**					120.88	<0.001*
1–3	2799	92.0	242	8.0		
4–7	2647	83.2	534	16.8		
≥8	48	73.9	17	26.1		

*Significant at *P* <0.05.

### Components and quality of ANC offered to pregnant women in Malawi

Of the 6,287 women, only 12.6% (n = 793) received quality ANC. Blood sample was collected from 95.0%(n = 5,975) of the participants. Urine samples were collected from 32.2%(n = 2,027) of the participants and 85.7% (n = 5,389) of the participants reported that their blood pressure was measured. In terms of preventative interventions, about 74.6% (n = 4,689) of the participants received the recommended two doses of tetanus vaccine and 48.0%(n = 3,019) received the recommended three or more doses of SP during ANC contacts as shown in Table 3.

**Table 3:** Components of ANC received during last pregnancy among women who had a live birth and received ANC at least once in the last two years prior to the survey in Malawi (n = 6,287).

Components of ANC	Frequency	%	95% CI
Blood pressure measured	5389	85.7	83.8–87.4
Blood sample taken	5975	95.0	94.2–95.7
Urine sample taken	2027	32.2	30.5–34.1
Given two or more tetanus injections	4689	74.6	73.1–76.0
Given three or more doses of antimalarial drugs	3019	48.0	46.2–49.9
Received all the five ANC components	793	12.6	11.4–13.9

### Factors associated with quality of ANC service among pregnant women in Malawi

Table 4 shows the results of the adjusted logistic regression. The results showed that education, parity, timing of ANC initiation and number of ANC contacts were significantly associated with quality of ANC. Women with only primary education level were 38% less likely to receive quality ANC compared to those with no formal education (AOR = 0.62; 95% CI = 0.48–0.82). Similarly, women who had given birth between 2 and 4 times were 19% less likely (AOR  =  0.81; 95% CI  =  0.66–0.98), while those with five or more births were 44% less likely (AOR  =  0.56; 95% CI  =  0.40–0.78) to receive quality ANC compared to their counterparts. On the other hand, women who started ANC within the first trimester had a 30% higher likelihood (AOR  =  1.30; 95% CI  =  1.10–1.53), those with four to seven ANC contacts had 2.1 higher odds (AOR  =  2.10; 95% CI  =  1.79–2.49), and those with at least eight ANC contacts had 3.4 times higher likelihood (AOR  =  3.40; 95% CI  =  1.90–6.09) of receiving quality ANC.

**Table 4:** Univariate and multivariate logistic regression analyses of factors associated with quality of ANC in Malawi.

Variables	UOR	95%CI	*p*-value	AOR	95%CI	*p*-value
**Age(years)**						
15–19	1			1		
20–34	0.88	0.72–1.07	0.190	0.92	0.72–1.17	0.496
35–49	0.59	0.45–0.79	<0.001*	0.85	0.58–1.25	0.405
**Area of residence**						
Urban	1			1		
Rural	0.67	0.55–0.82	<0.001*	0.80	0.64–1.02	0.066
**Region**						
Northern	1					
Central	0.93	0.73–1.18	0.544			
Southern	0.84	0.66–1.07	0.152			
**Marital status**						
Never married	1			1		
Currently married	0.87	0.65–1.18	0.372	1.07	0.78–1.48	0.673
Formerly married	0.65	0.46–0.94	0.020*	0.87	0.59–1.28	0.480
**Education**						
None	1			1		
Primary	0.71	0.55–0.92	0.009*	0.62	0.48–0.82	0.001*
Secondary and above	1.20	0.91–1.58	0.208	0.85	0.62–1.16	0.312
**Exposure to radio**						
Not all	1			1		
At least once a week	1.11	0.88–1.40	0.393	1.00	0.79–1.28	0.979
Less than once a week	1.28	1.09–1.50	0.003*	1.12	0.94–1.34	0.196
**Parity**						
1	1			1		
2–4	0.74	0.63–0.87	<0.001*	0.81	0.66–0.98	0.033*
≥5	0.48	0.38–0.61	<0.001*	0.56	0.40–0.78	0.001*
**Household wealth index**						
Poor	1			1		
Middle	0.99	0.80–1.23	0.962	0.93	0.74–1.15	0.485
Rich	1.29	1.10–1.52	0.002*	0.94	0.76–1.16	0.559
**Covered by health insurance**						
No	1			1		
Yes	2.33	1.03–5.30	0.043*	1.90	0.80–4.46	0.144
**Timing of first ANC visit**						
>3	1			1		
≤3 months	1.58	1.35–1.85	<0.001*	1.30	1.10–1.53	0.002*
**Number of ANC visits**						
1–3	1			1		
4–7	2.33	1.99–2.74		2.10	1.79–2.49	<0.001*
≥8	4.08	2.31–7.20	<0.001*	3.40	1.90–6.09	<0.001*

Note abbreviations: AOR: Adjusted odds ratio; UOR: Unadjusted odds ratio.

*Significant at *P* <0.05.

## Discussion

ANC is recognized as a crucial strategy for reducing maternal and neonatal mortality worldwide. The WHO emphasises that all pregnant women should receive quality ANC services to have positive pregnancy outcomes. These services include essential components of ANC such as urine test, blood pressure measurement, blood test and counseling on birth preparedness among others [4]. The purpose of the present study was to assess the quality of ANC services and explore associated factors in Malawi. The findings revealed that only 12.6% of women who had a live birth two years preceding the 2019–2020 MICS received quality ANC services. The findings show that timing of first ANC contact, number of ANC contacts, parity and education level were significantly associated with quality ANC.

In this study, we observed that 12.6% of pregnant women received quality ANC, a slight increase from the 11% reported in a previous study in Malawi [24]. Despite this improvement, the proportion remains low, suggesting that majority of pregnant women in Malawi do not receive the necessary care for detecting conditions that could negatively impact pregnancy outcomes. Our finding is consistent with studies conducted in East Africa [25], Cameroon [33], and Rwanda [34]. However, it is lower than the findings of studies conducted in Ethiopia [35], and Zambia [36]. The variations in the findings could stem from various factors, including differences in study settings, health systems and population demographics. Additionally, differences in study methodologies, sample sizes, and study durations could have contributed to the observed variations.

The present study revealed that pregnant women who initiated ANC within the first trimester and made at least four ANC contacts had higher likelihood of receiving quality ANC compared to their counterparts. Starting ANC within the first trimester helps women to receive quality care because they have more chances of interacting with health care providers who may provide all the contents of ANC than women who start ANC late. Furthermore, starting ANC early provides an opportunity for pregnant women to receive a wide range of health services that are important for improving the well-being of the mother and the unborn baby [37]. This finding is in agreement with existing literature [38,39]. Similarly, women who made four or more ANC contacts were more likely to receive quality ANC in this study. The more frequent a pregnant woman makes ANC contacts with health care providers, the higher the likelihood of receiving quality care. For instance, if a pregnant woman’s blood pressure is not checked during the first ANC contact, it is likely to be checked during subsequent contacts unlike a pregnant woman who does not visit health facility regularly. This finding is consistent with studies conducted in other countries [26,33,34,39]. Therefore, strengthening efforts to promote early ANC initiation and increasing the number of ANC contacts could significantly enhance the quality of ANC received by pregnant women in Malawi.

Consistent with existing studies [40–42], this study found that parity was negatively associated with quality of ANC. Multiparous women were less likely to receive quality ANC compared to primiparous women. There could be several reasons for this negative association. One possible explanation is that multiparous women may perceive themselves as experienced in pregnancy and childbirth, leading to a decreased perceived need for ANC services [43,44]. Consequently, they may not prioritize seeking ANC services or may believe they require less care than primiparous women. Another possible explanation is that health care providers may inadvertently provide less comprehensive ANC to multiparous women, assuming they are familiar with the process and do not require the same level of attention and services as first-time pregnant women. This could result in multiparous women receiving fewer contents of ANC or not having their specific needs adequately addressed during ANC visits. Furthermore, resource constraints within the health care system could contribute to disparities in receiving quality ANC based on parity. If healthcare facilities are overburdened or understaffed, multiparous women may receive less time and attention from health care providers during ANC visits compared to primiparous women. This finding highlights the importance of ensuring equitable access to adequate ANC services for all pregnant women, regardless of their parity status. Efforts to address this disparity may include targeted education and outreach programmes to promote the importance of ANC among multiparous women, as well as training health care providers to deliver adequate ANC to all pregnant women, regardless of their reproductive history.

Furthermore, this study found that education level was negatively associated with quality of antenatal care (ANC). Women with only primary level of education were less likely to receive quality ANC compared to those with no formal education. This finding is in contrast with the results of previous studies conducted in other countries [18,40,45]. This unexpected association may be attributed to several factors. Firstly, women with no education may have limited access to information about health care services, prompting them to prioritize seeking care and adhering more closely to health care recommendations. On the other hand, women with primary education may possess basic literacy skills but lack the health literacy necessary to fully comprehend the importance of ANC or actively engage with health care providers. Additionally, cultural beliefs may influence ANC utilization differently across education levels. These findings underscore the need for interventions that address broader societal factors, health literacy, and cultural norms to ensure equitable access to and utilization of quality ANC services across all education levels.

This study has some limitations which should be considered when interpreting the results. First, the data analysed were self–reported and prone to recall bias which may have resulted in misreporting of the contents of ANC pregnant women received. However, recall bias was minimised by limiting the analysis to women who had a live birth two years prior to the survey. Second, the analysis could not examine the effects of other variables of interest that may potentially be associated with quality of ANC due to lack of such explanatory variables in the MICS women dataset. Third, quality of ANC variable was constructed using five contents of ANC which were available in the MICS women dataset. As such, quality of ANC variable was not comprehensive as it was based on a limited number of recommended contents of ANC that should be provided to pregnant women during ANC visit. For example, other items for measuring quality of ANC including whether a pregnant woman received iron tablets or not during ANC contacts were not available in the MICS women dataset. Fourth, the cross-sectional design of the MICS restricts the interpretation of causality. Finally, this study utilized data collected prior to the roll out of the 2016 WHO ANC policy in Malawi. The policy was rolled out in Malawi in July 2019 [46]. Hence, future studies are warranted to evaluate any changes in the quality of ANC after the policy roll out in Malawi. Despite the above highlighted limitations, this study utilised nationally representative data, indicating that the findings possess external validity and can be generalized to the entire population of pregnant women in Malawi.

## Conclusion

The results of this study demonstrate that the quality of ANC provided to pregnant women in Malawi is substandard. The study has revealed that timing of first ANC contact, number of ANC contacts, parity and education are the significant factors associated with quality ANC. Women with only primary education were found to be less likely to receive quality antenatal care compared to those with no formal education. Similarly, women who had given birth between two and four times, as well as those with five or more births, were less likely to receive quality ANC compared to their counterparts. On the other hand, women who initiated ANC within the first trimester of pregnancy and attended a higher number of ANC contacts were more likely to receive quality ANC. These findings underscore the need for targeted interventions aimed at improving access to and utilization of ANC services, particularly among vulnerable populations such as women with lower education levels and higher parity. Strengthening efforts to promote early ANC initiation and increasing the number of ANC contacts could significantly enhance the quality of ANC that pregnant women receive, ultimately contributing to better maternal and child health outcomes in Malawi.

## Supporting information

S1 Checklist (DOCX)
